# Digital Mental Health Promotion Services for Youth: A Qualitative Study of Help-Seeking Through Mindhelper.dk

**DOI:** 10.2196/91017

**Published:** 2026-07-31

**Authors:** Amalie Oxholm Kusier, Caroline Høier Dalsgaard, Sofie Have Hoffmann, Lau Caspar Thygesen, Anna Paldam Folker

**Affiliations:** 1 National Institute of Public Health, The Faculty of Health Sciences, University of Southern Denmark, Studiestræde 6, Copenhagen, 1455, Denmark, 45 65507860

**Keywords:** digital mental health, youth mental health, help-seeking behavior, gender differences, digital letter box, mental health promotion, qualitative analysis

## Abstract

**Background:**

Young people increasingly experience mental health challenges and often turn to the internet for support. Self-guided digital mental health promotion services have become widely used resources for youth seeking help and guidance. These platforms offer accessible, anonymous support, yet little is known about the concerns young people articulate when engaging with them.

**Objective:**

This study aims to examine inquiries submitted to the digital letter box of Mindhelper.dk, Denmark’s most widely used digital mental health promotion service. Using qualitative analysis, the study aims to identify recurring themes in young people’s mental health concerns, explore gender differences in engagement, and generate insights to inform the development of more relevant and targeted digital self-help interventions.

**Methods:**

Using an inductive thematic approach informed by a constructivist-grounded theory coding framework, this study analyzes 2523 inquiries submitted to the Mindhelper digital letter box between March 2016 and August 2023. The dataset provides unsolicited first-person accounts from young people in moments of emotional vulnerability, offering insights into how mental health concerns are articulated in naturalistic settings.

**Results:**

The analysis identifies 17 recurring themes reflecting the mental health challenges young people seek help for. These were grouped into 3 overarching analytical categories: Social Relationships and Social Contexts, Emotional Life, and Body and Illness, with the first 2 dominating the material. Prominent themes included Sociality, Love Life, Unease, Self-doubt and Insecurity, and Seeking Support. Across genders, inquiries frequently focused on social relationships, particularly Sociality and Love Life. However, girls were markedly overrepresented among users, while only minor gender differences were observed in the distribution of themes.

**Conclusions:**

The findings suggest that young people’s mental health concerns are closely tied to everyday developmental and relational challenges rather than severe psychopathology alone. Digital letter box services may capture early expressions of distress that might not otherwise reach formal support systems. This highlights the preventive potential of such services and the value of using self-initiated digital data to inform the development of relevant digital mental health support and better understand how young people articulate and act on emerging mental health concerns.

## Introduction

A substantial proportion of young people in Denmark report poor mental health. This is particularly pronounced among young women, with 34.8% of women aged 16‐24 years reporting poor mental health in the 2023 Danish National Health Survey, compared with 16.0% among men in the same age group [1]. A recent assessment of well-being in Denmark further indicates that more than 50% of young women aged 16‐24 years report high levels of stress, compared with 31,6% among young men in the same age group [2]. This challenge is not unique to Denmark; globally, youth mental health trends indicate a substantial increase in issues such as anxiety, psychological distress, self-harm, suicide, and depressive symptoms [3,4]. According to the Lancet Psychiatry Commission, mental ill health now accounts for at least 45% of the overall burden of disease among individuals aged 10‐24 years, and suicide remains the fourth leading cause of death among those aged 15‐29 years worldwide [5]. These figures highlight the significant global impact of mental health problems among young people.

Adolescence and emerging adulthood (the latter typically defined as ages 18‐25 years) represent particularly vulnerable periods due to rapid physical, cognitive, emotional, and social development [6]. Poor mental health during these formative years can have severe long-term consequences [7,8]. For example, one study found that adolescents with poor mental well-being had significantly higher risks of mental illness, self-harm and suicide attempts, alcohol and substance abuse, interpersonal violence, and all-cause mortality than those with better mental well-being [9]. Emerging adulthood is also a critical window for the onset of mental disorders, with 63%‐75% of mental illnesses first appearing before the age of 25 years [5]. This emphasizes the need for preventive strategies during this developmental stage.

In response to these challenges, digital mental health interventions have emerged as a promising approach [5,10,11]. Digital mental health interventions refer to mental health care delivered via digital technologies, and they can take a variety of forms depending on the target population, purpose, and content of the intervention, the technology used, and the degree of human interaction involved [12]. Research indicates that such interventions have considerable potential to promote mental health, prevent distress, and support the treatment of mental illness. As young people are among the highest users of the internet and digital technologies, these interventions offer advantages such as accessibility, flexibility, and broad reach, making them well suited to address some of the limitations observed in traditional youth mental health services [5]. However, the potential of digital mental health services to promote well-being among young people is not without challenges. Evidence suggests that the level of engagement with digital interventions is critical for achieving positive mental health outcomes [13]. Yet, engagement is influenced by a range of facilitators and barriers that determine whether young people adopt and continue using these services [14].

To ensure that digital mental health support services are effective, it is essential that they are designed with content perceived as relevant and useful by young people. A systematic review by Ho et al [15] identified perceived fit and perceived usefulness as key facilitators of engagement with web-based mental health interventions for young people. Perceived usefulness was enhanced when interventions provided informative and actionable content, while content that was repetitive, overly simple, unspecific, or already known acted as barriers. Similarly, perceived fit was strengthened when interventions were age-appropriate, relevant, and culturally sensitive. Personalization—such as individualized interaction and feedback—further improved perceived fit, whereas lack of personalization and irrelevance (eg, cultural mismatch) reduced engagement. Findings from Borghouts et al [16] reinforce these conclusions, emphasizing that participants were more likely to engage when they perceived the intervention to be useful and a good fit for them. Furthermore, a review has shown that research on youth digital mental health interventions is primarily based on young women, leaving a significant gap in knowledge about how young men engage with and experience these interventions [17]. Understanding these gendered aspects is essential to ensure that digital mental health services are inclusive and meet the needs of all young people.

Building on this, this study seeks to contribute to the field by providing insight into the issues young people seek help for when experiencing mental distress. This is achieved by examining inquiries submitted to a digital letter box in Denmark’s most widely used digital mental health promotion services, Mindhelper.dk. Specifically, the study aims to (1) identify recurring themes in young people’s inquiries about mental health and well-being, (2) examine how gender may shape these experiences in the context of engaging with a self-guided digital platform, and (3) generate insights to inform the design of more relevant and targeted digital self-help interventions for young people. Through qualitative analysis of letter box inquiries, this study offers valuable knowledge about the mental health challenges faced by Danish youth and their engagement with digital support services.

## Methods

This section presents the methodological framework of the study. To situate the analysis, we first describe the empirical context, followed by a detailed account of the data and ethical considerations. Finally, we outline the analytical strategy applied in the study.

### Empirical Context

Mindhelper.dk was launched in 2016 and is now the largest public digital youth mental health–promoting service in Denmark with approximately 1 million visitors a year. Mindhelper is a self-guided service that aims to provide young people in Denmark with knowledge, advice, and tools on a wide range of mental health and well-being topics, including anxiety, depression, dating, and friendship problems, self-harm, and suicidal thoughts. The platform offers online courses, papers, videos, and guidance on where to seek help offline, as well as a digital letter box for personalized advice. In addition to its website, Mindhelper also provides support through an app with a focus on reducing stress, pressure, or anxiety.

Mindhelper primarily targets young people aged 13‐25 years, but usage data show that the platform is also widely accessed by children younger than 13 years, young adults older than 25 years, and adults such as parents and professionals (eg, schoolteachers and student counselors). All services at Mindhelper are free, self-guided, and fully anonymous, requiring no user account.

Mindhelper was developed through a collaboration between the Centre for Digital Psychiatry (CEDIP) and 4 Danish municipalities. CEDIP oversees ongoing operations and content development, which is cocreated with young people through a youth network and a panel of 12 members who contribute to testing, feedback, and idea development. Since 2019, Mindhelper has been permanently funded by Denmark’s 5 regions, and in 2025, national government funding was added.

### The Data Material

This study explores young people’s inquiries to the Mindhelper letter box over a period of nearly 8 years, covering inquiries submitted between 2016 and 2023. Since 2016, young people have been able to write anonymously to the digital letter box and receive a response within 10 days from psychology students employed at CEDIP. All inquiries and responses are published on Mindhelper.dk, allowing users to read and benefit from each other’s experiences. The only editing occurs when personally identifiable information is included, in which case the inquiry is anonymized. There is no censorship of content. Inquiries can be accessed via free-text search using subject-specific keywords or browsed through thematic categories available on the website.

Over time, a few changes have been made to the letter box. When the letter box was launched, young people were required to state their gender (male or female) and age (13‐20 years) when submitting inquiries based on set categories. In 2021, the gender requirement was removed, and, in summer 2024, the age category was expanded to 13‐25 years. When submitting inquiries, young people can write up to 1200 characters, and the date and time are automatically entered.

The letter box archive represents a unique empirical dataset, offering rare access to anonymous, first-person accounts from young people written in moments of emotional vulnerability. Unlike retrospective interviews or researcher-generated data, these inquiries are unsolicited and self-initiated, providing immediate and authentic expressions of mental health concerns. This makes the material particularly valuable for understanding youth experiences of distress and well-being as they unfold. At the same time, it is important to note that, as a digitally mediated dataset, the material is influenced by the platform’s affordances and constraints, including its written format, character limits, and the characteristics of its user base.

The dataset analyzed includes all inquiries submitted to the letter box between March 4, 2016, and August 7, 2023, comprising a total of 2523 inquiries. The data extraction was conducted by CEDIP and included only the inquiries from the young people–not the associated responses–to ensure that the analysis was not influenced by the content of the replies. Additionally, the analysis did not take into account Mindhelper’s thematic categorization of the inquiries.

### Ethical Considerations

This study is registered and approved by the University of Southern Denmark in accordance with the Data Protection Regulation and the General Data Protection Regulation (EU) 2016/679 and has additionally been approved by the University’s Research Ethics Committee (approval ID 23/24293). The analyzed material consists of anonymous inquiries submitted to the letter box of a digital mental health promotion platform. No compensation was provided, as users submitted inquiries voluntarily as part of their use of the platform. All inquiries were anonymized before researcher access and were already publicly available. Although users consented to having their posts published, they were not informed that the content might later be used for research, which raises an ethical consideration about secondary use of data. We assess the risk of harm as minimal because the data were anonymized and originally shared in a public forum. Obtaining informed consent was not feasible, as submissions were anonymous.

### Protection of Anonymity in Quotations

However, several measures were applied to balance the use of illustrative quotations with the protection of contributor anonymity. First, all quotations were translated from Danish to English using an interpretive, meaning-based translation process rather than a literal translation. This constituted an additional analytical transformation and reduced traceability. Second, the majority of quotations were shortened to retain analytical relevance while avoiding unnecessary detail that could increase identifiability. In addition, quotations were deliberately selected to illustrate broader themes, shared feelings, or general experiences reflected across multiple inquiries, rather than highly specific or personally identifying narratives that also appeared in the material. Third, although inquiries on the platform were publicly associated with unique identification numbers, these identifiers were deliberately omitted from all quotations and descriptions in the manuscript to prevent direct linkage to the original submissions. Overall, this approach was consistent with principles for the responsible use of publicly available, anonymized data.

### Analysis Strategy

#### Coding Framework

The analysis followed an inductive thematic approach, informed by Charmaz’s constructivist grounded theory as a pragmatic coding framework, to examine patterns of meaning across the 2523 inquiries [18-20]. While guided by constructivist grounded theory principles, the aim was not to generate a formal grounded theory or explanatory model but to use iterative coding strategies to develop analytically grounded themes and categories. Coding followed 2 main phases described by Charmaz: initial coding, involving close reading of each word, line, or segment of data to remain open to multiple possible interpretations, and focused coding, which uses the most significant or frequent initial codes to sort, synthesize, and organize larger segments of the dataset [19]. These phases were operationalized across 3 coding rounds: round 1 (initial coding), and rounds 2 and 3 (focused coding), as described in the following sections.

Given the size of the dataset, we used NVivo (Lumivero), a qualitative analysis software, to facilitate data storage, coding, and comparison [18]. To ensure feasibility, the first author (AOK) conducted a pilot coding on a small sample (n=49 inquiries), focusing on the technical aspects of accessing and organizing the material and enabling comparison across the dataset. This confirmed NVivo as a suitable tool for managing the data. The test coding did not involve engagement with the thematic content, ensuring consistency with the inductive and grounded approach applied in the study.

#### Coding Round 1: Initial Open Coding

All inquiries were read and coded by the first author (AOK). The aim of the initial open coding round was to identify topics, descriptions, and statements that contributed to a nuanced understanding of why the young person contacted the letter box. Following the grounded theory principle of line-by-line coding, each relevant segment was assigned a code, and a single inquiry could contain multiple codes, as short statements or excerpts often addressed several points [19]. The coding process remained close to the data, using simple and precise codes, as outlined in the codebook (Multimedia Appendix 1). Importantly, coding was kept open, allowing new codes to be created as new perspectives emerged. In addition to assigning short code titles, a code description was developed for each code.

In the open coding round, gender, age, and the electronically registered submission time and date were specified for each inquiry. For inquiries where gender was not stated—since it was not a requirement—“unknown” was entered, unless the content of the inquiry explicitly indicated gender (eg, “I am a 16-year-old girl”), thus relying solely on explicit, self-reported statements of gender and avoiding interpretive attribution. This would have applied equally to all explicitly stated gender identities, whether binary or nonbinary.

Gender and age were also adjusted if the information explicitly provided in the inquiry differed from the predefined categories, in which case the text always took analytical precedence. Such adjustments were particularly common in relation to age, as some young people stated that they were younger than 13 years or older than 20 years. Similarly, gender categorizations were guided exclusively by how young people described themselves in the inquiry text. If a young person had explicitly stated a gender identity other than girl or boy, this would have been recorded accordingly; however, no inquiries included such explicit self-description.

Among the inquiries included in the analysis, it was not possible to identify the gender of the young person in 455 inquiries. It should be emphasized that the unspecified gender category should not be interpreted as reflecting a particular gender identity or group. Rather, it solely indicates that the young person did not disclose their gender, regardless of whether their gender identity is binary, nonbinary, or otherwise.

#### Coding Round 2: Focused Coding and Refinement of Codes

In the second coding round, we moved toward focused coding, aiming to synthesize and explain larger segments of data [19]. At this stage, we sought to ensure consistency in the content of the codes and comparability between the inquiries assigned to each code, as the goal was to determine the adequacy of the codes. The first author (AOK) reread all coded data within each of the 115 codes, and any inquiry that did not align with the overall content of its assigned code was recoded. Similarly, code titles and descriptions were revised when necessary to better reflect their content and maintain proximity to the data. Codes were deleted if they became irrelevant due to recoding or merged when they overlapped with other codes. Code descriptions were actively used as analytical reference points during this process to assess whether the inquiries grouped under each code consistently reflected the defined content of the code. Where discrepancies were identified, codes and their descriptions were iteratively revised to maintain conceptual coherence and proximity to the data. After reviewing each code, frequently used words were searched across the remaining material to identify any potentially overlooked inquiries. This process involved comparing data with data and data with codes, which helped refine and strengthen the coding structure. Following this focused coding round, the number of codes was reduced to 101 (for a complete overview of the codes, see Multimedia Appendix 1).

#### Coding Round 3: Analytical Theme Formation

In the third and final round of coding, the 101 empirically generated codes were grouped into 17 distinct analytical themes. This step marks another move away from initial coding, which fractured the data into discrete segments, toward reassembling the material into a coherent whole. The focus is on constructing an analytical narrative that brings the emerging categories together in a meaningful and connected way [19]. The first author (AOK) drafted the initial themes, which were then discussed jointly with all the study’s authors. Through this iterative process, the final themes and their labeling were iteratively refined and defined. To provide an additional overview of the thematic directions in the material, the 17 themes were further grouped into 3 overarching analytical categories: Social Relationships and Social Contexts, Emotional Life, and Body and Illness (Multimedia Appendix 1).

Following the coding rounds and the identification of themes, exploratory cross-tabulations were conducted between themes, as well as between themes and gender, age, and time of submission (month and year) using NVivo; only patterns deemed analytically meaningful are reported in the results. The unit of analysis was the individual inquiry; accordingly, the reported counts represent the number of inquiries associated with each theme, rather than population-level distributions of concerns.

In the presentation of the 17 identified themes in the Results section, selected quotes are included to support the thematic analysis. These quotes were chosen based on their capacity to vividly exemplify the thematic patterns identified. The aim was to foreground excerpts that best articulate the meaning and scope of each theme, thereby supporting the analytical claims made and providing insight into how each theme is grounded in the data.

### Exclusion of Inquiries

During the 3 rounds of coding, inquiries were continuously excluded. Of the 2523 inquiries, 93 were excluded, primarily because they fell outside the scope of the letter box service—for instance, inquiries from young people seeking assistance with school assignments. Inquiries submitted by parents concerning their children’s well-being were also excluded, as the study focuses exclusively on inquiries submitted by young people themselves. Inquiries that were incomprehensible were likewise excluded. Additionally, 19 inquiries could not be categorized under broader themes due to their highly individual content and are therefore not included in the thematic analysis. The final thematic structure is based on 2411 inquiries.

## Results

This section presents the main findings, including identified themes, intersections between themes, and gendered patterns.

### Thematic Analysis

Textbox 1 presents the 17 identified themes, organized across 3 analytical categories: Social Relationships and Social Context, Emotional Life, and Body and Illness. Each theme includes an integrated analytic description that captures recurring patterns in the data. For ease of presentation, the themes will hereafter be referred to using their short title shown in italics.

**Figure 1.**
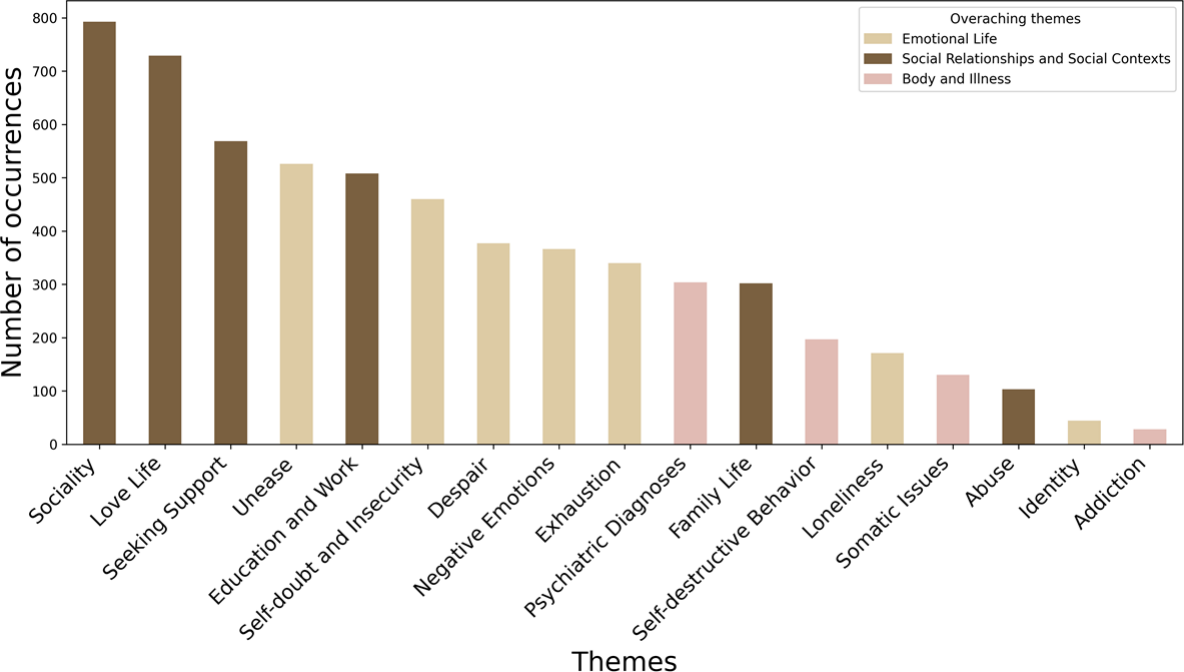
The distribution of inquiries across 17 identified themes.

As illustrated in Figure 1, young people predominantly write about themes within the overarching category Social Relationships and Social Contexts when reaching out to the letter box service. However, a notable number of inquiries also touch upon topics related to complex emotions, as reflected in the themes grouped under the category Emotional Life. Themes within the overarching category Body and Illness appear less frequently in the inquiries, with the notable exception of the theme Psychiatric Diagnoses, which stands out as more prominent.

Textbox 1. **Identified themes with integrated analytic descriptions.**

**Social Relationships and Social Contexts**

- Sociality: navigating social relationships
- Love Life: navigating romantic relationships and feelings
- Seeking Support (represents a crosscutting theme related to help-seeking behavior rather than a distinct concern or emotional issue): attempts to seek support that are often unmet
- Education and Work: experiencing educational distress and uncertainty
- Family Life: experiencing strained family relationships
- Abuse: experiences of violence and harm in relationships

**Emotional Life**

- Unease: experiencing persistent anxiety and worry
- Self-Doubt and Insecurity: negative self-evaluation
- Despair: experiences of hopelessness, emptiness, and loss of meaning
- Negative Emotions: low and fluctuating mood
- Exhaustion: persistent tiredness and feeling overwhelmed
- Loneliness: feeling alone and excluded from others
- Identity: struggling to form identity

**Body and Illness**

- Psychiatric Diagnoses: experiences of and seeking clarification about psychiatric diagnoses
- Self-Destructive Behavior: engaging in harmful practices
- Somatic Issues: bodily symptoms, experiences, and concerns
- Addiction: experiences and concerns related to addictive behaviors

Figure 1 shows the distribution of the 2411 inquiries across the identified themes and 3 analytical categories.

The analyses indicate that inquiries to the letter box come from children and young people aged between 8 and 28 years, with the median age across themes ranging from 15 to 17.5 years. Those writing about topics such as *Love Life*, *Psychiatric Diagnoses*, and *Somatic Issues* tended to be slightly older than individuals addressing other themes. In contrast, inquiries related to *Self-Destructive Behavior* were generally submitted by younger individuals compared with the remaining themes. Furthermore, cross-tabulations between themes and time of submission did not reveal any notable patterns.

### Social Relationships and Social Contexts

Social relationships and the social contexts that young people navigate throughout adolescence play a significant role in their inquiries to the digital letter box. Under this analytical category, we present 6 themes that represent some of the most frequently addressed topics in young people’s inquiries.

With 793 inquiries, *Sociality* emerges as the most addressed theme, reflecting how young people navigate social relationships. Nearly half of the inquiries within this theme involve friendships, for example, fears of losing friends, broken friendships, conflicts, or feeling misunderstood or unwanted, as the following quote illustrates:

*[...] I don’t really talk to many people my own age, and I haven’t spent time with anyone outside of school or work in the past year, except for my partner. Honestly, I don’t know what to do and just feel like crying. When I do try to take the initiative to arrange something with friends or acquaintances, it never ends up happening. I generally just feel rejected and unaccepted. It’s like I’m stuck in a bubble all by myself.*

[Boy, aged 17 years]

Some describe dilemmas such as romantic feelings for friends, worries about friends, or comparing themselves to others. Other submissions reflect more broadly on social life—how they act in social settings, expectations to be social, or doubts about their social skills, for example:

*I am 20 years old and do not feel that I am doing enough “typical youth things.” I often feel more mature than my peers. I therefore feel that I have too few friends and do not know what to spend my time on after work... [...]*.

[Gender unspecified, aged 20 years]

Some young people seek advice on becoming more social, while others express a desire to withdraw. Many describe insecurity in social situations, such as fear of judgment, saying the wrong thing, or feeling awkward. Others mention shyness, introversion, or being withdrawn. A number of young people also write about concerns of close relationships—family, partners, or friends—where young people seek advice on how to support someone or cope with being close to someone with, for example, mental illness, as expressed by a 13-year-old girl: “My friend has been going through a difficult time lately, and I would like to help him with it. [...].” Finally, some write about social relationships or contexts that have had a particularly negative impact, such as encounters with bullying, experiences of betrayal and mistrust, facing cultural practices within the family, and dealing with difficulties forming close connections.

*Love Life* is also a central theme with 729 inquiries, highlighting how young people navigate romantic relationships and feelings. These include experiences of being in love, having a crush, or flirting with someone. One submission reads:

*I’m a 13-year-old boy. I’ve met a girl—she’s really sweet, but I don’t know her very well. I think I might have a crush on her, but I’m not sure what that feels like since I’ve never had a girlfriend before. I don’t know if we’re a good match, because she goes to a different school than I do, and she’s 12. I don’t know if she likes me back, and I’m afraid of embarrassing myself. How can I tell if she likes me—can I sense it from the way she talks or looks at me? Thanks in advance for your help*.

[Boy, aged 13 years]

While such experiences can be positive, they can also involve dilemmas, such as falling for a friend, someone with a different sexual orientation, or someone who does not reciprocate the feelings. Additionally, young people write about heartbreak and ex-partners, as one shares: “I desperately need help. My boyfriend broke up with me very suddenly, which now makes me feel like my life has come to a standstill [...]” [Girl, aged 17 years]. Numerous inquiries concern challenges or dilemmas related to romantic relationships with partners including insecurity, jealousy, being apart from their partner, doubts about the relationship’s future, and conflicts between romantic partners and friends or family. Few young people write about broader worries in love life, such as how to handle specific romantic situations, worries about finding love, or meeting expectations around love: “[...] I have realized that I am probably afraid of love. I am afraid of being let down. I am afraid of being hurt [...]” [Girl, aged 16 years]. Intimate experiences and sexuality are rarely mentioned, but the few inquiries that do address these topics describe issues such as sex in relationships, sexual expectations, reflections on sexual debut, and coming out or exploring one’s sexual identity.

Represented by 569 inquiries, the theme *Seeking Support* points to how young people attempt to seek support but are often not met with the help they need. Rather than reflecting a specific domain of concern, this theme captures how young people navigate help-seeking processes across different types of issues. In the inquiries, young people reflect on or describe experiences, attempts, or actual interactions of seeking help or support related to worries or mental health struggles, most often involving reaching out to partners, friends, or parents. While some young people report positive experiences when reaching out to others, many instead receive limited or insufficient support. Others describe explicitly negative experiences, such as being rejected or dismissed by those they turned to, as the quote demonstrates:

*I feel like I can’t turn to anyone, and I’ve been feeling really bad lately. Everywhere I read, it says you should talk to someone about it, but what if you don’t have anyone to talk to at all? When I try to talk to my mom, we end up arguing because she doesn’t understand me and places really high expectations on me. My dad and I don't have a close relationship. [...]*

[Gender unspecified, aged 15 years]

Several express reservation about seeking help due to fear of mental health stigma and the shame it generates, and a few mention lacking a support network entirely—prompting them to turn to the letter box instead. Experiences of seeking professional help, such as from general practitioners, psychologists, or services such as Headspace and BørneTelefonen (The Children’s Helpline), are also shared in the inquiries:

*[...] I have been going to a psychologist. She didn’t help me, and she seemed indifferent to me and my problems just like everyone else is, indifferent...So what can I do now? [...]*

[Girl, aged 15 years]

These encounters are often described as inadequate or disappointing, leading them to seek support through the Mindhelper letter box. Others ask for advice on whether or how to approach professional support. Additionally, several inquiries mention interactions with teachers—ranging from helpful conversations to unsuccessful attempts to get support. Finally, a few young people express a general difficulty in talking about emotions or opening up to others.

A total of 508 inquiries is included under the theme *Education and Work* capturing experiences of educational distress and uncertainty. Nearly half describe educational distress—often due to feelings of insecurity, loneliness, or experiences of exclusion from their class, as expressed by the 17-year-old girl: “I am terrified that I will be left out again in group work. […].” A few young people mention both supportive and difficult teacher relations. Many inquiries concern academic performance, including descriptions of pressure to achieve good grades or difficulties keeping up academically, for example: “My grades are dropping, and it really stresses me out a lot. [...]” [Girl, aged 14 years]. Challenges such as giving presentations, participating in group work, or engaging in classroom activities are also mentioned. Additionally, a few young people write about difficulties with concentration in school. Several young people express thoughts about dropping out, changing their educational path, or switching schools, indicating that youth is a time marked by transitions that often lead to worry and reflection. This is illustrated by a young person who states:

*I need your help. I’m in a situation where I’m unsure whether I’ve chosen the right education or the right place for my apprenticeship. My motivation isn’t what it used to be when I first started, and I no longer look forward to going to work. I don’t feel safe or comfortable at my workplace the way I think I should. [...] I’m training to become a baker, but the way people speak there doesn’t really match the kind of person I am. I’m quiet, shy, and introverted, and the industry is much tougher and harsher than I expected. What should I do?*

[Girl, aged 19 years]

These concerns are also reflected in inquiries about choosing an education, taking a gap year, or starting at a new educational institution. A few inquiries concern work or part-time jobs, describing pressure, poor conditions, low energy, or workplace well-being. Some also seek advice on job opportunities.

*Family Life* is addressed in 302 inquiries, illustrating experiences of strained family relationships. Young people describe their relationship with parents or family dynamics, shaped by events such as divorce. Some describe specific conflicts or disagreements with parents, while others express general distress at home, saying that they find it difficult to be there:

*I can’t be around my parents for more than a couple of hours without us starting to argue. Not a day goes by without them making me cry. Normally, I’m a strong person, but I can’t handle being torn down every day, and I no longer want to live at home. [...]*

[Boy, aged 15 years]

A few mention problematic parental behavior, including dynamics marked by violence or growing up with alcohol abuse by 1 or both parents. Some inquiries also include descriptions of sibling relationships, which can be either positive or negative—for example, concerns about siblings or reflections on how sibling dynamics affect the family as a whole.

The least frequent theme within the category of Social Relationships and Social Contexts, *Abuse*, appears in 103 inquiries, pointing to experiences of violence and harm in relationships. Young people report experiences of physical, psychological, or sexual violence, as illustrated by the account of a young person:

*[...] At home, I’m having some problems...My dad...I’m afraid of him...But I’m also very stubborn and often talk back. But he becomes very violent when things don’t go his way...He yells loudly, grabs us (me and my little brother), and sometimes pushes us. [...]*

[Girl, aged 16 years]

Over half concern physical abuse, including violence in romantic relationships or violence observed between parents. The inquiries also include descriptions of sexual abuse, rape, or incest. Others describe psychological or verbal abuse, and a few reflect on having inflicted psychological harm on others.

### Emotional Life

A large proportion of young people’s inquiries to the letter box concern their emotional life, reflected in 7 distinct themes. *Unease* emerges as the most prominent theme within the emotional category, represented by 528 inquiries, reflecting experiences of persistent anxiety and worry. Young people describe unease in various ways, often explicitly referring to anxiety or panic attacks, for example: “I have struggled with anxiety since I was 6 years old, but it has never been diagnosed. [...]” [Girl, aged 15 years]. Others report uncontrollable racing thoughts, overwhelming worries, or a tendency to overthink everything, for example, worries about the future, or intrusive fears related to death or illness, as expressed in the following excerpt from an enquiry:

*I can’t be in myself anymore, I have far too many thoughts that I can’t manage. I overthink EVERYTHING, to the point where it hurts. All kinds of thoughts are spinning around in my head. It’s so exhausting, and it affects my everyday life. I can’t even feel happy anymore, because I'm constantly thinking about everything. [...]*

[Girl, aged 16 years]

In some cases, unease is expressed through physical symptoms resembling panic attacks, such as heart palpitations, shortness of breath, sweating, or dizziness. In a limited number of inquiries, young people describe a sense of unreality, such as feeling detached from their body or being unable to distinguish between dream and reality.

With 460 inquiries, the theme of *Self-doubt and Insecurity* emerges as a central concern revealing patterns of negative self-evaluation among the young people writing to the letter box. The young people express this by thoughts of not feeling good enough, feeling worthless, or being generally unsure of oneself. For some, this is aimed at their body and appearance, with statements such as feeling ugly or fat, as expressed in the following quote:

*I’m a 15-year-old girl writing here because I’m really unhappy with my body and appearance. Lately, I’ve become truly dependent on food and have been eating large amounts outside of meals—often in secret because it feels so shameful. I just feel ugly and stupid. My family sees me in a very negative light, which I guess is understandable, but it’s also exhausting*.

[Girl, aged 15 years]

A small number of inquiries also describe a pervasive perfectionism, rooted in high expectations of oneself and a fear of failure. In other cases, young people explicitly state that they struggle with low self-esteem or lack of confidence. Among 377 inquiries, young people express profound *Despair*, reflected in experiences of hopelessness, emptiness, and loss of meaning. Over half of the inquiries within this theme mention suicidal thoughts, while others describe a sense of detachment—likened to, for example, carrying a black hole inside.

*I’ve been feeling empty for a while. Especially in the evenings, I feel like I have nothing to live for. I find it hard to concentrate at school. Sometimes I feel okay, but other times I feel absolutely terrible. Sometimes I just wish I could disappear. Of course I want to live, but I also want to feel good. I’m afraid to tell my parents, even though I know it’s one of the best things I could do. I’m also scared to say it out loud, mostly because I don’t have a reason to be sad. But still, I feel really awful. Is it normal to cry without having a reason?*

[Gender unspecified, aged 13 years]

A recurring statement is the loss of motivation and desire, whether for everyday tasks, former interests, or pursuing education or work. Some express a wish to escape—physically by leaving everything behind or mentally by becoming invisible or disappearing. For others, it takes the form of hopelessness and the belief that nothing will ever improve.

*Negative Emotions* such as feeling sad, depressed, or downcast are reflected in 366 inquiries, where low and fluctuating mood frequently emerges. Many young people describe a persistent sense of emotional distress, often articulated as a lack of joy, a constant urge to cry, or crying frequently, which is exemplified by a 20-year-old young woman, who opens her inquiry by writing: “I have been feeling sad and down for quite some time. Without being able to say for sure, I’m afraid I might be developing depression. [...].”

While sadness dominates this theme, a few inquiries express anger or frustration, sometimes through descriptions of shouting or screaming, as a 17-year-old girl writes in her inquiry: “[...] I also get angry and irritated with people more quickly, and that makes me even more angry. [...].” Some young people seek advice on managing overwhelming emotions or mood swings. This theme also includes experiences of grief and anticipatory grief.

*Exhaustion* is a recurring theme in 340 inquiries, described as constant fatigue or feeling drained of energy. Sleep problems are also common, including difficulty falling asleep or poor sleep quality, for example: “[...] I have trouble sleeping. I often lie for several hours with my eyes closed. [...]” [Boy, aged 13 years]. Additionally, some report feeling stressed, pressured, or overwhelmed in their daily lives. These experiences are clearly illustrated in the following inquiry:

*I feel constantly down, sad, and incredibly tired all the time. I usually bike 14 km on weekdays, but I can't cope with it, and I'm just really sad. I get stressed and angry quickly, and I find it hard to tolerate many things, which makes me easily irritated with people. What could be wrong with me? Or what can I do to feel better?*

[Girl, aged 16 years]

The theme of *Loneliness* is based on 171 inquiries, with loneliness expressed both directly and indirectly in young people’s accounts of feeling lonely, alone, or experiencing life as lonely. While several young people explicitly mention loneliness, others express it through statements about often being alone, as seen in the words of a 15-year-old who begins their inquiry with: “I feel very left out, wrong, and alone. [...].”

The final theme within Emotional Life concerns *Identity* and appears in 44 inquiries, where young people struggle to form a sense of identity. Most involve young people reflecting on their interests and hobbies as part of shaping their identity. One young person describes her struggle with finding purpose:

*I just have this problem that I don’t have any interests or hobbies to spend my time on. I have a boyfriend and a job, and when I’m not working or with my boyfriend, I simply don’t know what to do. As a child, I tried a whole range of extracurricular activities—everything from handball to cooking and crocheting—but nothing ever caught my interest. I have no talents, and I’m neither creative, musical, nor anything like that. TV series and movies don’t really appeal to me, and books can’t hold my attention either. Because I don’t have anything I’m passionate about or spend my time on, I feel empty inside*.

[Girl, aged 19 years]

A few describe compulsive lying to close relations—sometimes, for example, about sexual experiences—which have spiraled out of control. Others express a sense of identity crisis, feeling disconnected from who they once were.

### Body and Illness

While 4 themes concerning Body and Illness have been identified, their significance appears limited within the overall narrative of young people’s inquiries to the letter box. However, several young people write about *Psychiatric Diagnoses*, constituting the largest theme within this category, with 304 inquiries. Thus, although the category Body and Illness itself is relatively small, the theme *Psychiatric Diagnoses* ranks as the tenth most prevalent across the 17 identified themes, as previously shown in Figure 1. This theme includes both young people’s experiences with psychiatric diagnoses and their desire to clarify whether they might have one. Depression is the most frequently described condition, with young people sharing current or past experiences of living with depression, and many seeking advice and information about whether they might be experiencing symptoms. This is illustrated in the words of a young person who writes:

*I’m unsure whether I’m just going through a rough patch or if I’m starting to develop depression. I feel really down, drained of energy, and I don’t find joy in the things that usually make me happy. [...]*

[Girl, aged 20 years]

In addition to depression, 13 other psychiatric diagnoses are mentioned in the inquiries, with some of the most frequently cited being eating disorders, autism, obsessive-compulsive disorder, and attention-deficit/hyperactivity disorder.

The theme of *Self-Destructive Behavior* is identified in 197 inquiries, where young people engage in harmful practices, primarily covering accounts of current or past self-harming behavior such as cutting, scratching the skin, or hitting themselves. One young person shares: “I really can’t take it anymore...Self-harm and suicidal thoughts are taking up so much space, and I just feel completely awful about myself. [...]” [Girl, aged 15 years]. Additionally, several young people express having a disturbed relationship with food, which may manifest in both their thoughts and behaviors.

A total of 132 inquiries to the letter box concern *Somatic Issues* encompassing bodily symptoms, experiences, and concerns. About half describe recurring physical symptoms linked to psychological distress, such as stomach pain, headaches, physical discomfort, tension, dizziness, nausea, and heart palpitations.

*[...] I have so many thoughts that just keep going around in circles. Thoughts like: Why do I always do everything wrong, why didn’t I say anything, I’m fat, I should lose weight, etc. These thoughts also often make me feel nauseous, and I feel like I’m about to throw up just from the thought of having to eat something. [...]*

[Girl, aged 15 years]

Other inquiries reflect the impact of living with physical illness or injury on mental health and well-being. Additionally, several young people ask questions about the body, including reactions to various conditions, menstruation, pregnancy, contraception, sexually transmitted infections, diet, and exercise.

The final theme related to Body and Illness is the smallest in the letter box analysis, with 28 identified inquiries concerning *Addiction* focusing on young people’s experiences and concerns related to addictive behaviors. Over half involve the use of alcohol, cannabis, or harder drugs as the young person writes about in his inquiry:

*I've lost my entire circle of friends from childhood and school to hash or because I got kicked out of school. [...] Now I mostly sit alone from morning to evening and smoke myself numb. [...] I feel alone with everything and can’t really see a way out...Help plz*.

[Boy, aged 19 years]

While young people do not always describe their use as addiction, the extent of substance use is central to the problems they seek help for. Others write about addiction-related issues such as dependency on gaming, exercise, or shopping.

### Intersection of Themes

**Figure 2.**
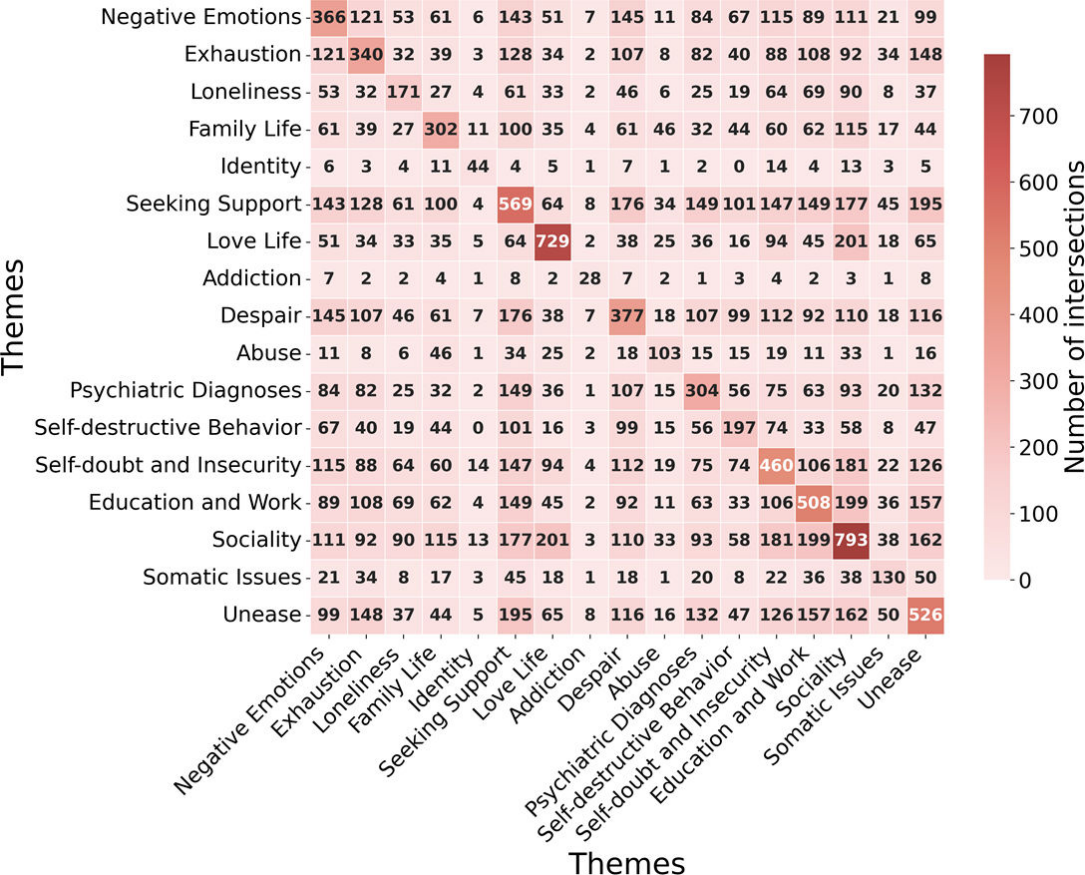
Intersection of themes.

This section explores the intersections between the 17 identified themes. The heatmap (Figure 2) shows how the themes are connected, with darker areas indicating stronger overlaps between themes. By presenting the data as a heatmap, we gain a clearer understanding of the complexity and interrelated nature of young people’s concerns.

The central role of social relationships in young people’s well-being is confirmed by examining the 3 most frequent combinations of 2 themes. A total of 201 inquiries from young people address both the themes *Love Life* and *Sociality*. These entries often revolve around dilemmas related to romantic feelings and friendships, as the 14-year-old girl expresses it in the title of her inquiry: “I’m in love with my friend, but she’s not into girls,*”* or issues involving partners and friends, as expressed by a 13-year-old girl who writes: “I have a friend who can’t keep her hands off my boyfriend.”

The combination of the themes *Sociality* and *Education and Work* is also frequently represented in young people’s inquiries (n=199). These inquiries often include descriptions of social challenges within the school context and how such difficulties negatively impact their overall school well-being, as one young person puts it:

*I’m a 14-year-old girl in 8th grade. I don’t really fit in with my class, and I really want to. I feel left out during events and I’m not really part of the conversations. [...] And I really want to have more friends in my class and maybe be part of the community. But I don’t know how*.

[Girl, aged 14 years]

The third most common thematic combination is found in the pairing *Seeking Support* and *Unease* (n=195). A significant proportion of the inquiries within this theme combination reflect young people’s experiences with anxiety, while also expressing efforts to seek professional help or posing questions about how to access such support. This is exemplified in the following inquiry:

*I think I’m struggling with social anxiety. I’ve never been formally diagnosed, but I’ve had a few panic attacks. I generally feel very uncomfortable in large groups of people, and I break out all over my body when I’m at parties, in my school’s cafeteria, or even just walking down the street. I’m fairly aware of my situation and I know what to do when I experience these “attacks” and the spots on my skin. Still, I’m tired of how much it affects my everyday life, and that’s why I wanted to ask what you think I should do about it. Should I start seeing a psychologist and talk about it, even though I’m quite self-aware? Or should I just accept that this is how my daily life is going to be?*

[Girl, aged 18 years]

Furthermore, Figure 2 highlights that most inquiries to the digital letter box involve multiple themes within a single inquiry, with intersections appearing across nearly all 17 themes. Notably, the heatmap reveals that the theme *Seeking Support* is frequently addressed in conjunction with other themes, indicating that it rarely stands alone. This theme captures young people’s experiences of navigating help-seeking processes in response to distress, functioning as a crosscutting dimension rather than expressing a specific concern or emotional issue, as is the case with the other 16 themes. Accordingly, it underscores the potential of a digital letter box as a space for outreach when traditional support networks feel inaccessible. The only theme combination not represented is between *Identity* and *Self-Destructive Behavior*, marked with a zero in the corresponding square to indicate that no inquiries addressed both themes.

### Gender Patterns Across Themes

Table 1 presents the gender distribution across themes, highlighting a notable gender imbalance in young people’s use of the letter box, with girls being overrepresented. Although this overall trend is clear, only slight variations appear when comparing gender distribution across individual themes. For instance, *Love Life* was the most frequently addressed theme among boys (105/286, 36.7%) compared with girls (499/1670, 29.9%) and those with unspecified gender (125/455, 27.5%). Conversely, *Sociality* was most prevalent among girls (567/1670, 34.0%) and the unspecified gender category (130/455, 28.6%), although it was also common among boys (96/455, 33.6%). Girls (416/1670, 24.9%) and individuals with unspecified gender (105/455, 23.1%) wrote more often about *Seeking Support* than boys (48/286, 16.8%). Similarly, for *Education and Work*, girls (366/1670, 21.9%) and those with unspecified gender (96/455, 21.1%) addressed these themes more frequently than boys (46/286, 16.1%).

**Table 1.** Gender patterns across themes.

Themes	Inquiries^a^					
	Girls, n (%)		Boys, n (%)		Unspecified gender, n (%)	
Sociality	567 (34.0)		96 (33.6)		130 (28.6)	
Love Life	499 (29.9)		105 (36.7)		125 (27.5)	
Seeking Support	416 (24.9)		48 (16.8)		105 (23.1)	
Education and Work	366 (21.9)		46 (16.1)		96 (21.1)	
Family Life	205 (12.3)		30 (10.5)		67 (14.7)	
Abuse	68 (4.1)		14 (4.9)		21 (4.6)	
Unease	377 (22.6)		43 (15.0)		108 (23.7)	
Self-Doubt and Insecurity	315 (18.9)		50 (17.5)		95 (20.9)	
Despair	254 (15.2)		43 (15.0)		80 (17.6)	
Negative Emotions	289 (17.3)		22 (7.7)		55 (12.1)	
Exhaustion	253 (15.1)		26 (9.1)		61 (13.4)	
Loneliness	119 (7.1)		18 (6.3)		34 (7.5)	
Identity	28 (1.7)		9 (3.1)		7 (1.5)	
Psychiatric Diagnoses	229 (13.7)		25 (8.7)		50 (11.0)	
Self-Destructive Behavior	154 (9.2)		12 (4.2)		31 (6.8)	
Somatic Issues	88 (5.3)		14 (4.9)		30 (6.6)	
Addiction	9 (0.5)		12 (4.2)		7 (1.5)	
Total	1670 (100)		286 (100)		455 (100)	

a A single inquiry may appear under multiple themes.

Small variations were also observed across gender categories within themes related to emotional life. Young people with unspecified gender (108/455, 23.7%) and girls (377/1670, 22.6%) were more likely to address topics related to *Unease* than boys (43/286, 15.0%). Similarly, experiences with *Negative Emotions* were more frequently described by girls (289/1670, 17.3%) and those with unspecified gender (55/455, 12.1%) than by boys (22/286, 7.7%). Mentions of *Exhaustion* were also more common among girls (253/1670, 15.1%) and the unspecified gender category (61/455, 13.4%) than among boys (26/286, 9.1%).

For themes related to body and illness, the greatest variation was observed between boys and girls. Girls were considerably more likely to write about *Self-Destructive Behavior* (154/1670, 9.1%) than boys (12/286, 4.2%), while individuals with unspecified gender fell in between (31/455, 6.8%). Likewise, boys addressed subjects related to the theme *Addiction* more frequently (12/286, 4.2%) than girls (9/1670, 0.5%), with the unspecified gender category again positioned between the two (7/455, 1.5%).

## Discussion

### Principal Findings

This study analyzed inquiries submitted to the digital letter box of Mindhelper, Denmark’s most widely used digital mental health promotion service, to identify key mental health themes and gender differences in engagement. A thematic analysis revealed 17 themes across 3 overarching categories— Social Relationships and Social Contexts, Emotional Life, and Body and Illness.

With some of the most salient themes centering on *Sociality* and *Love Life*, our findings confirm adolescence as a socially sensitive stage [6,21] and extend existing knowledge by showing that distress rarely occurs in isolation. Instead, it is deeply entangled with relational contexts such as friendships, romantic relationships, and family life [22,23]. These patterns may help inform how digital support services are structured to better reflect and respond to interconnected user needs.

A central insight emerging from this study is that digital help-seeking often occurs at the intersection between unmet needs in informal support networks and barriers to formal care. While previous research has emphasized the importance of social networks for youth help-seeking and conceptualized it as a socially embedded process [24-28], our findings suggest that some young people turn to digital platforms precisely when these networks are experienced as insufficient, inaccessible, or unresponsive. This shift carries both potential and limitations. On the one hand, digital letter boxes provide a low-threshold, anonymous entry point to support, enabling young people to articulate concerns that might otherwise remain unexpressed [29,30]. On the other hand, help-seeking outside established social relationships may risk reinforcing isolation or limiting opportunities for sustained support, particularly given the one-off and text-based nature of such services. The findings suggest that digital mental health services should be designed not only as stand-alone support but also as bridges to existing social and professional resources, with particular attention to users who lack supportive networks.

Second, our analysis highlights gendered engagement patterns without sharp divides. While social relationships are important for both boys and girls, boys more frequently address *Love Life*, whereas girls more often write about friendships, broadly social life, and themes related to Emotional Life. While girls dominate overall volume, boys’ inquiries often cluster around behavioral expressions such as addiction and abuse, suggesting that they may be more likely to externalize distress through behaviors such as substance use, a pattern noted in previous research on gendered expressions of distress [31].

These patterns prompt a closer look at help-seeking behavior. Girls turning to the digital letter box for advice far more often than boys may reflect a higher prevalence of distress among Danish girls, as documented in national health surveys [5], but it may also indicate that boys are less inclined to seek help. Previous research has identified gendered barriers to help-seeking among young men [32-35]. For instance, a review of 31 studies found that 27 identified masculine attitudes—such as the need to appear strong, preference for self-management, and viewing help-seeking as weakness—as barriers to seeking support [32]. While the review covered youth help-seeking and health care access broadly, most studies focused on mental health (n=17) and sexual health (n=10). Although evidence suggests that understandings of masculinity and related behaviors influence perceptions of mental health and help-seeking [31], several scholars stress the importance of incorporating broader contextual factors when examining gender differences in help-seeking behavior. For instance, studies have shown that ethnicity, education, and age also influence help-seeking tendencies [36-38]. McKenzie et al [39] also underline the importance of adopting an intersectional perspective to better understand men’s experiences of mental illness stigma, noting that factors such as homophobia and racism can shape these experiences. Moreover, gender differences in help-seeking appear to diminish at higher levels of psychological distress, which may suggest that men are less likely than women to recognize mild symptoms of mental distress [40-42]. Furthermore, research has highlighted the role of social network quality in shaping help-seeking behavior [43,44].

Building on this, the role of the digital context should be further considered in studies of gender differences in help-seeking and engagement within mental health services. Digital features such as anonymity and accessibility may shape some of the patterns observed between genders. Importantly, a review study highlights that attitudes and preferences—such as trust and distrust in health care providers, beliefs about the necessity or effectiveness of care, perceived dissimilarity with providers, preferences for self-reliance, and concerns about anonymity or other potential negative consequences—are strong predictors of help-seeking among young people [45]. This suggests that research on gendered barriers to help-seeking should consider not only the individual factors but also the broader context of service provision, including whether services are delivered digitally. In line with other scholars, we therefore point to the need for models of help-seeking and engagement that incorporate these contextual factors to gain a more comprehensive understanding of gendered digital mental health behavior and to inform the development of designs that more effectively appeal to boys [41,46,47].

Analyses such as the present one are important because they enable the systematic identification of emerging help-seeking needs among young people. These needs might otherwise go unnoticed and yet are essential for developing relevant support content. At the same time, it is necessary to consider the rapidly evolving landscape of digital mental health services driven by technological developments. Over the course of this study, major changes occurred that may influence young people’s help-seeking behavior and use of digital services such as letter boxes. In late 2022, ChatGPT was launched, and by 2025, artificial intelligence–generated content became a dominant feature of Google’s search results, potentially introducing new sources for youth seeking mental health advice. Previous studies, conducted before these developments, show that text-based queries via search engines remain the most common online help-seeking approach among young people [30,48]. This reliance on search engines underscores how artificial intelligence integration may reshape the information young people encounter, creating new challenges for developers to ensure access to professional, evidence-based resources. These developments underscore the need for future research to examine their implications for youth help-seeking behavior and engagement with digital mental health platforms.

### Strengths and Limitations

While this study offers valuable insight, it is important to acknowledge both its methodological strengths and limitations. A key strength lies in its unique data foundation, providing rare access to young people’s real-time expressions of mental health challenges, rather than retrospective accounts. The anonymous digital context enables open engagement with sensitive and stigmatized topics, supporting more candid insights into youth experiences. Additionally, the large and temporally broad dataset allows for a comprehensive view of evolving challenges young people face.

Despite the study’s strengths, several limitations should be acknowledged. Each submission to the Mindhelper letter box provides a one-time snapshot of a young person’s mental health concerns, without the possibility of follow-up or contextual elaboration beyond what the individual chooses to express. Moreover, the material reflects a specific context: young people who choose to engage with this type of digital service. Consequently, the sample may be biased toward those who are willing and able to seek help through digital platforms. In addition, written communication is central on a digital platform, which means the format requires users to be able to read and write, potentially excluding youth with limited literacy skills. However, no formal writing standards are imposed, which is reflected in the data, occasionally marked by linguistic ambiguity.

Finally, attention should be drawn to the analytical process. The coding was conducted by a single researcher, which may have introduced some bias. However, the systematic and transparent approach, combined with reflexive engagement and author group involvement, supports the credibility of the analysis. In addition, changes in gender categories on Mindhelper introduced limitations for the gender-related analyses. Initially, binary categories allowed some insight into differences between boys’ and girls’ mental health struggles, although this approach excluded nonbinary youth and may have discouraged nonbinary self-identification. Removing gender categories entirely improved inclusivity but restricted the ability to examine how gender may shape mental health. Future research should seek methods that capture gender diversity while enabling meaningful analysis.

### Conclusions

Seventeen themes emerged, reflecting the diverse concerns young people seek advice for, with social relationships and emotional experiences constituting central domains. Gender differences were observed in the extent of engagement, with girls more frequently using the digital letter box than boys, while only minor variations appeared in the distribution of themes across genders. Taken together, the findings suggest that many of the concerns young people raise are closely tied to everyday developmental and relational challenges rather than severe psychopathology alone. The digital letter box thus captures a broader spectrum of lived experience, including early signs of distress that may not otherwise reach formal systems. This highlights both the preventive potential of such services and the value of using self-initiated digital data to better understand how young people articulate and act on emerging mental health concerns.

## Supplementary material

10.2196/91017 Multimedia Appendix 1 Codebook.

## Abbreviations

CEDIP: Centre for Digital Psychiatry
