# Supplementary material for: Digital Mental Health Promotion Services for Youth: A Qualitative Study of Help-Seeking Through Mindhelper.dk
Source: JMIR Hum Factors. 2026 Jul 31;13:e91017. doi: 10.2196/91017 (PMC13427080; doi:10.2196/91017)
Supplement: Multimedia Appendix 1 [file humanfactors-v13-e91017-s001.docx]

# **Multimedia Appendix 1 Codebook**

*Coding note: Individual inquiries could be assigned multiple codes within the same theme; thus, code-level frequencies may exceed the total number of inquiries reported for each theme.*

## Category: Social Relationships and Social Contexts

| **Theme: Sociality – navigating social relationships (n=793)** | | | | | | | | | | |
| --- | --- | --- | --- | --- | --- | --- | --- | --- | --- | --- |
| **Code** | | | **Code description** | | | **N = inquiries** | | |  |  |
| Friendships | | | Concerns about losing friends or having lost friendships, experiencing challenges or conflicts with friends, or feeling that friends do not want to engage or understand you. Includes dilemmas in friendships, such as being in love with a friend, worrying about friends, or comparing yourself to them | | | 382 | | |  |  |
| Social life | | | Thoughts about their social life and how they behave in social situations, including expectations about how they should act socially and their social skills. Covers both the desire to be more social and join social communities, as well as the wish not to be social. | | | 195 | | |  |  |
| Insecurity in social situations | | | Fear of what others think of you, saying or doing something wrong in social settings, feeling socially awkward, feeling insecure in social situations, or having difficulty talking to others. | | | 116 | | |  |  |
| Concerns about close relationships | | | Worries about a close relationship, such as a family member, partner, or friend, due to issues like mental illness, self-harm, or physical illness. Often includes seeking advice on how to help the person. May also cover experiences of going through a period filled with concerns about close relationships, for example due to physical illness. | | | 70 | | |  |  |
| Mental illness in close relationships | | | Descriptions of a close relation such as a partner, friend, sibling, or parent having or having had a mental illness (for example, during the young person’s childhood). | | | 64 | | |  |  |
| Bullying | | | Descriptions of being bullied, exposed to bullying behavior, or past experiences with bullying that still have a negative impact. | | | 62 | | |  |  |
| Experience of betrayal and mistrust | | | Descriptions of feeling betrayed by parents, friends, partners, or the healthcare system. Also includes descriptions of lacking trust in others. | | | 44 | | |  |  |
| Shyness | | | Descriptions of being shy or struggling with shyness. | | | 41 | | |  |  |
| Introverted and reserved | | | Descriptions of being introverted and/or reserved. | | | 31 | | |  |  |
| Islam and cultural expectations | | | Descriptions of issues related to Islamic cultural practices | | | 11 | | |  |  |
| Attachment difficulties | | | Descriptions of being unable to form attachments, commit to others, or struggling to build relationships or let people in. | | | 9 | | |  |  |
| **Theme: Love Life – navigating romantic relationships and feelings (n=729)** | | | | | | | | | |  |
| **Code** | | **Code description** | | | **N = inquiries** | | | | |  |
| Crushes | | Descriptions of being in love, having a crush, or flirting. Includes dilemmas such as being attracted to a friend or someone with a different sexuality, as well as unrequited love. | | | 304 | | | | |  |
| Romantic relationships | | Descriptions of issues or questions related to romantic relationships. This can include insecurity in the relationship, jealousy, missing the partner, concerns about whether the relationship will last, worries about the partner, or conflicts between the partner and friends/family. | | | 247 | | | | |  |
| Breakups and ex-partners | | Descriptions of problems related to ex-partners or heartbreak, and seeking advice on how to move on after a breakup. | | | 122 | | | | |  |
| Love | | Descriptions of challenges related to love, advice on handling specific situations, or concerns about finding love or meeting certain expectations about love. | | | 74 | | | | |  |
| Sex and kissing | | Descriptions of issues related to sex, for example in a relationship or a “friends with benefits” situation where sex is central. Also includes expectations about sexual debut and kissing. | | | 56 | | | | |  |
| Sexuality | | Descriptions of issues related to sexuality, such as considering coming out as homosexual, reflecting on one’s own sexuality, or experiences where sexuality plays a role, for example falling in love with a friend who is not homosexual. | | | 30 | | | | |  |
|  | | | | | | | | | |  |
| **Theme: Seeking Support – attempts to seek support that are often unmet (n=569)** | | | | | | | | | |  |
| **Code** | | **Code description** | | | **N = inquiries** | | | | |  |
| Reaching out to one’s network | | Descriptions of experiences reaching out to a network such as a partner, friends, or family about personal problems or concerns. Includes cases where reaching out did not help or was a negative experience (e.g., rejection), as well as situations where the person has no one to reach out to due to a small network. | | | 358 | | | | |  |
| Experiences with professional help or seeking professional help | | Descriptions of current or past experiences seeking or receiving professional help from, for example, a doctor, psychiatrist, psychologist, or services like Headspace or the Children’s Helpline. Also includes questions about where or how to access professional help | | | 230 | | | | |  |
| Seeking or receiving help from a teacher | | Descriptions of talking to a teacher about personal concerns, receiving help from a teacher, or trying to tell a teacher without getting help. | | | 81 | | | | |  |
| Difficulty talking about feelings and opening up | | Descriptions of struggling to talk about feelings, communicate how one feels, or open up to others. | | | 35 | | | | |  |
| **Theme: Education and Work – experiencing educational distress and uncertainty (n=508)** | | | | | | | | | |  |
| **Code** | **Code description** | | | **N = inquiries** | | | |  |  |  |
| Well-being in school or education | Descriptions of general distress in school or education, such as feeling unsafe, lonely, or excluded in class. | | | 240 | | | |  |  |  |
| Academic performance | Descriptions related to performance in education, such as grades, struggling to keep up with lessons, feeling unsuccessful in school, and challenges with presenting, participating in group work, or engaging in class | | | 149 | | | |  |  |  |
| Dropping out or changing school | Descriptions of considering dropping out of education, changing school or program. Also includes experiences of having dropped out and how that decision affects them, as well as past school changes. | | | 114 | | | |  |  |  |
| Educational transitions | Descriptions of thoughts or concerns related to educational choices, gap years, or starting at new institutions. | | | 113 | | | |  |  |  |
| Work and part-time jobs | Descriptions of well-being at work, feeling pressured at work, lacking energy to go to work, or experiencing a poor work environment. Also includes questions about job opportunities. | | | 46 | | | |  |  |  |
| Relationship with teachers | Descriptions of positive or negative relationships with teachers. | | | 25 | | | |  |  |  |
| Concentration at school | Descriptions of having difficulty concentrating in school or asking for advice on improving concentration. | | | 16 | | | |  |  |  |
| **Theme: Family Life – experiencing strained family relationships (n=302)** | | | | | | | | | |  |
| **Code** | **Code description** | | | **N = inquiries** | | | |  |  |  |
| Relationships with parents | Descriptions of the relationship with parents or family dynamics, such as divorce. Includes conflicts or disagreements with parents, having a difficult family, feeling that “things are hard at home,” or poor parental behavior such as violence. Also covers worries about a parent or feeling guilty toward parents. | | | 275 | | | |  |  |  |
| Sibling relationships | Descriptions of relationships with siblings, concerns about siblings, or how siblings affect family dynamics. | | | 65 | | | |  |  |  |
| Parental alcoholism | Descriptions of alcohol abuse by one or both parents or growing up in a home with alcoholics. | | | 14 | | | |  |  |  |
| Adoption | Issues related to being adopted. | | | 3 | | | |  |  |  |
| **Theme: Abuse – experiences of violence and harm in relationships (n=103)** | | | | | | |  |  |  |  |
| **Code** | **Code description** | | | **N = inquiries** | | | |  |  |  |
| Physical violence | Descriptions of having been subjected to violence, witnessing violent relationships between parents, committing violence against others, or being in an abusive relationship | | | 56 | | | |  |  |  |
| Sexual abuse and rape | Descriptions of rape, sexual assault, and sexual abuse. | | | 28 | | | |  |  |  |
| Psychological violence | Descriptions of being subjected to psychological violence, verbal abuse, or committing psychological violence against others. | | | 19 | | | |  |  |  |
| Incestuous relationships and abuse | Descriptions of incestuous relationships and abuse, or questions related to these. | | | 10 | | | |  |  |  |
| Violation and boundary crossing | Descriptions of being violated or having personal boundaries crossed. | | | 9 | | | |  |  |  |

## Category: Emotional Life

| **Theme: Unease – experiencing persistent anxiety and worry (n=528)** | | |
| --- | --- | --- |
| **Code** | **Code description** | **N = inquiries** |
| Anxiety | Descriptions of having anxiety, anxiety attacks, or questions about the anxiety diagnosis. Includes various forms of anxiety, such as performance anxiety, fear of death, exam anxiety, social anxiety, and panic anxiety. | 305 |
| Racing thoughts and overthinking | Descriptions of experiencing racing thoughts, overwhelming worries, or overthinking things | 160 |
| Future | Descriptions of concerns about the future, such as what they want to do with their life or how illness might affect their future. Also includes descriptions of hopes and goals for the future. | 59 |
| (Panic) attacks | Descriptions of panic attacks or episodes described with physical and psychological symptoms such as heart palpitations, shortness of breath, dizziness, sweating, chest pain, feeling choked, or tearfulness. Does not include cases where anxiety attacks or panic anxiety are explicitly mentioned. | 43 |
| Being afraid | Descriptions of feeling afraid of everything, having constant fear, or being scared all the time. | 29 |
| Obsessive and distressing thoughts | Descriptions of having obsessive thoughts or thoughts that feel unpleasant or confusing. | 28 |
| Feeling of unreality | Descriptions of feeling that everything or the world seems unreal, not knowing if one is in a dream or reality, or feeling disconnected from one’s body. | 26 |
| General feeling of nervousness | Descriptions of a general sense of nervousness. | 23 |
| Fear of dying or death | Descriptions of being afraid of dying or being terminally ill, including thoughts about death and descriptions of death anxiety. | 19 |
| Feeling restless | Descriptions of feeling restless. | 9 |
| **Theme: Self-doubt and Insecurity – negative self-evaluation (n=460)** | | |
| **Code** | **Code description** | **N = inquiries** |
| Not feeling good enough | Descriptions of feeling inadequate, worthless, dissatisfied with oneself, or insecure. Also includes self-critical thoughts, self-hate, or talking down to oneself. | 184 |
| Appearance and body | Descriptions of insecurities or negative thoughts about one’s body or appearance, such as feeling ugly or fat. | 118 |
| Low self-esteem or confidence | Descriptions of having low self-esteem or low confidence. | 104 |
| Guilt, shame, self-blame, and bad conscience | Descriptions of feeling guilty, ashamed, blaming oneself, or having a bad conscience, for example about one’s own distress. | 99 |
| Nobody likes me | Descriptions of feeling or worrying that nobody wants you, nobody likes you, or that everyone hates you. | 52 |
| Perfectionism and fear of failure | Descriptions of being perfectionistic, setting high standards for oneself, and fearing failure. | 22 |
| Feeling different | Descriptions of feeling different, strange, weird, abnormal, or wrong, as well as experiences of not being understood by others because of being or thinking differently. | 21 |
| Insecurity about specific abilities | Descriptions of feeling unsure about being able to manage or succeed in specific tasks, such as getting a driver’s license or expressing oneself verbally. | 6 |
| **Theme: Despair – experiences of hopelessness, emptiness, and loss of meaning(n=377)** | | |
| **Code** | **Code description** | **N = inquiries** |
| Suicidal thoughts | Descriptions of having suicidal thoughts, wanting to die, not wanting to be here anymore, or feeling undeserving of life. Also includes past suicidal thoughts or suicide attempts. | 198 |
| Sense of meaninglessness and emptiness | Descriptions of feeling life is meaningless, feeling empty inside, having a “black hole,” experiencing indifference, or feeling emotionally numb. Also includes reflections on the meaning of life. | 127 |
| Lack of motivation and desire | Descriptions of not feeling motivated to do things, losing interest or desire. This can include everyday activities, things they used to enjoy, or completing education and/or work. | 68 |
| Wanting to escape | Descriptions of wanting to get away from people, everything, disappear, become invisible, hide away, take a break, or not wanting to be here anymore. Also includes descriptions of wanting to be physically away, for example in another country. | 42 |
| Lack of hope for improvement | Descriptions of having no hope of getting better, that things will improve, or that help is possible. Also includes feeling hopeless or that life is hopeless. | 35 |
| Hating life or being tired of life | Descriptions of hating life or feeling tired of life. | 17 |
| Existential loss of control | Descriptions of feeling no control over life, that everything is collapsing, having no foundation, or that life is slipping through their fingers. | 8 |
| **Theme: Negative emotions – low and fluctuating mood (n=366)** | | |
| **Code** | **Code description** | **N = inquiries** |
| Feeling sad | Descriptions of feeling sad, depressed, down, or upset. Can also be expressed as lacking joy in life, wanting to cry all the time, or being able to cry constantly. | 294 |
| Feeling angry or mad | Descriptions of being angry or mad, experiencing anger, having outbursts, being irritable, aggressive, bitter, filled with hatred, or behaviors such as shouting or screaming. | 48 |
| Managing emotions or emotional reactions | Descriptions of having emotions or emotional reactions such as intense temper, tearfulness, or strong jealousy that the young person wants help to control. | 39 |
| Mood swings | Descriptions of experiencing mood swings or having moods that change constantly. | 20 |
| Experiencing grief and anticipatory grief | Descriptions of grief and loss of close persons such as family members or friends. Includes anticipatory grief related to terminally ill parents, as well as grief over losing pets. | 19 |
| **Theme: Exhaustion – persistent tiredness and feeling overwhelmed (n=340)** | | |
| **Code** | **Code description** | **N = inquiries** |
| Tiredness or lack of energy | Descriptions of feeling constantly tired, exhausted, drained of energy, or having no strength to cope. | 160 |
| Sleep problems | Descriptions of being unable to sleep or fall asleep, having sleep difficulties, trouble sleeping, or sleeping poorly. | 131 |
| Stress, pressure, and busyness | Descriptions of feeling stressed about things or “stressing,” feeling stressed, pressured, or having too much to do. | 126 |
| **Theme: Loneliness – feeling alone and excluded from others (n=171)** | | |
| **Code** | **Code description** | **N = inquiries** |
| Loneliness | Descriptions of feeling lonely, past or current experiences of loneliness, or perceiving situations as lonely. | 125 |
| Being or feeling alone | Descriptions of feeling alone, feeling alone with a worry, feeling completely alone, fearing being alone, or feeling abandoned. | 60 |
| **Theme: Identity – struggling to form identity (n=44)** | | |
| **Code** | **Code description** | **N = inquiries** |
| Hobbies and interests | Descriptions of issues related to hobbies or the desire to have more hobbies or interests. | 32 |
| Lying | Descriptions of lying a lot or wanting to lie. That the lying is getting out of control and being hard to manage. Does not refer to small lies or lying about specific situations. | 6 |
| Identity crisis | Descriptions of being in an identity crisis or not knowing oneself. | 6 |

## Category: Body and Illness

| **Theme: Psychiatric Diagnoses – experiences of and seeking clarification about psychiatric diagnoses (n=304)** | | | |
| --- | --- | --- | --- |
| **Code** | **Code description** | **N = inquiries** |  |
| Depression | Descriptions of having depression or past experiences with depression. Also includes questions about the diagnosis and symptoms of depression or depressive traits. | 191 |  |
| Eating disorder | Descriptions of having an eating disorder, either current or previously diagnosed, or questions about whether one has an eating disorder. | 45 |  |
| Autism | Descriptions of having autism or questions about the diagnosis. | 21 |  |
| OCD | Descriptions of having OCD or questions about the diagnosis. | 21 |  |
| ADHD | Descriptions of having ADHD, being medicated for ADHD, or questions about the diagnosis. | 17 |  |
| Personality disorder | Descriptions of having a personality disorder, questions about the diagnosis, or whether one might have a personality disorder. Includes various types, such as borderline. | 14 |  |
| Hearing voices or seeing visions | Descriptions of hearing voices or seeing visions (hallucinations). | 14 |  |
| ADD | Descriptions of having ADD, being medicated for ADD, or questions about the diagnosis. | 9 |  |
| PTSD | Descriptions of having PTSD or questions about the diagnosis. | 9 |  |
| Schizophrenia | Descriptions of having schizophrenia, being assessed for schizophrenia, or questions about the diagnosis. | 9 |  |
| Phobias | Descriptions of having phobias or believing one suffers from a phobia, e.g., emetophobia. | 6 |  |
| Misophonia | Descriptions of having misophonia, suspecting misophonia, or questions about the diagnosis. | 2 |  |
| Bipolar disorder | Descriptions of having bipolar disorder or questions about the diagnosis. | 1 |  |
| Tourette’s | Descriptions of having Tourette’s. | 1 |  |
| **Theme: Self-destructive Behavior – engaging in harmful practices (n=197)** | | | |
| **Code** | **Code description** | **N = inquiries** |  |
| Self-harm | Descriptions of engaging in self-harm or “cutting,” as well as past experiences of self-harm. Includes behaviors such as scratching the skin, hitting oneself, or cutting oneself. | 164 |  |
| Disturbed relationship with food | Descriptions of having a problematic relationship with food, which may manifest in thoughts and/or behaviors. Does not necessarily explicitly mention eating disorder. | 40 |  |
| **Theme: Somatic Issues – bodily symptoms, experiences, and concerns (n=132)** | | | |
| **Code** | **Code description** | **N = inquiries** |  |
| Physical symptoms of psychological distress | Descriptions of continuous or recurring physical symptoms of distress, such as stomach pain, headaches, physical discomfort, tension, restlessness in the body, shaking, dizziness, nausea, and heart palpitations. | 71 |  |
| Physical illness and injuries | Descriptions of how chronic illness, physical illness, or physical injuries affect mental health. | 24 |  |
| Questions about the body | Descriptions of or questions about the body’s reactions to various things. | 14 |  |
| Menstruation | Descriptions of or questions about having menstruation. | 9 |  |
| Body, diet, and exercise | Descriptions of questions or thoughts related to diet, exercise, and energy levels. | 7 |  |
| Contraception and sexually transmitted diseases | Descriptions of experiences with or questions about contraception and/or sexually transmitted diseases. | 6 |  |
| Pregnancy | Descriptions of concerns related to pregnancy or being pregnant. | 5 |  |
| **Theme: Addiction – experiences and concerns related to addictive behaviors (n=28)** | | | |
| **Code** | **Code description** | **N = inquiries** |  |
| (Mis)use of substances | Descriptions of using substances (e.g., alcohol, cannabis, or harder drugs). This can be described either as misuse or simply use. | 19 |  |
| Addiction to video games | Descriptions of or questions about addiction to video games. | 6 |  |
| Addiction to exercise | Descriptions of or questions about addiction to exercise. | 2 |  |
| Addiction to shopping | Descriptions of or questions about addiction to exercise. | 1 |  |
